# Disorders of sex development (DSD) web-based information: quality survey of DSD team websites

**DOI:** 10.1186/s13633-019-0065-x

**Published:** 2019-05-28

**Authors:** Michelle M. Ernst, Diane Chen, Kim Kennedy, Tess Jewell, Afiya Sajwani, Carmel Foley, David E. Sandberg, in collaboration with the DSD-TRN Psychosocial Workgroup and Accord Alliance

**Affiliations:** 1 Department of Pediatrics, University of Cincinnati College of Medicine, Cincinnati, Ohio 45229 USA; 2 Disorders of Sex Development Center, Cincinnati Children’s Hospital Medical Center, 3333 Burnet Ave., ML 3015, Cincinnati, OH 45229 USA; 3 Pritzker Department of Psychiatry and Behavioral Sciences, Ann & Robert H. Lurie Children’s Hospital of Chicago, 225 E. Chicago Ave., Box 161B, Chicago, IL 60611 USA; 4 Potocsnak Family Division of Adolescent and Young Adult Medicine, Ann & Robert H. Lurie Children’s Hospital of Chicago, 225 E. Chicago Ave., Box 161B, Chicago, IL 60611 USA; 5 Departments of Psychiatry and Behavioral Sciences, and Pediatrics, Northwestern University Feinberg School of Medicine, 446 E. Ontario Street, Chicago, IL 60611 USA; 6 Oberlin College, 173 W. Lorain St., Oberlin, OH 44074 USA; 7 Hofstra Northwell School of Medicine, 420 Lakeville Rd, Suite 110, New Hyde Park, NY 11042 USA; 8 Department of Pediatrics and Susan B Meister Child Health Evaluation and Research (CHEAR) Center, University of Michigan Medical School, 300 North Ingalls St., Rm 6C23, Ann Arbor, MI 48109 USA

**Keywords:** Health literacy, Internet health information, Disorder of sex development

## Abstract

**Objectives:**

Consumers rely on online health information, particularly for unusual conditions. Disorders of Sex Development (DSD) are complex with some aspects of care controversial. Accurate web-based DSD information is essential for decision-making, but the quality has not been rigorously evaluated. The purpose of the present study was to assess the quality of online health information related to DSD presented by 12 pediatric institutions comprising the NIH-sponsored DSD-Translational Research Network (DSD-TRN).

**Methods:**

DSD-TRN sites identified team webpages, then we identified linked webpages. We also used each institution search engine to search common DSD terms. We assessed webpages using validated tools: the Simple Measure of Gobbledygook (SMOG) determined reading level, the Patient Education Materials Assessment Tool (PEMAT) evaluated content for understandability and actionability, and the DISCERN tool assessed treatment decision-making information (for hormone replacement and surgery). We developed a “Completeness” measure which assessed the presence of information on 25 DSD topics.

**Results:**

The SMOG reading level of webpages was at or above high-school grade level. Mean (SD) PEMAT understandability score for Team Pages and Team Links was 68% (6%); on average these pages met less than 70% of the understandability criteria. Mean (SD) PEMAT actionability score was 23% (20%); few patient actions were identified. The DISCERN tool determined that the quality of information related to hormone treatment and to surgery was poor. Sites’ webpages covered 12–56% of the items on our Completeness measure.

**Conclusions:**

Quality of DSD online content was poor, and would be improved by using a variety of strategies, such as simplifying word choice, using visual aids, highlighting actions patients can take and acknowledging areas of uncertainty. For complex conditions such as DSD, high-quality web-based information is essential to empower patients (and caregiver proxies), particularly when aspects of care are controversial.

## Introduction

The internet is a primary source of health information for patients and families. Of the more than 80% of U.S. adults who report using the internet (as of 2012), 72% indicate they have used it for online health information [1]. Individuals in the midst of a health crisis [2] or with impaired access to appropriate care [3] are more likely to seek online information, as are consumers accessing information related to unusual or stigmatizing conditions [4]. Consumers seek information both for themselves and for others [5] and look for educational information on diagnostic features of conditions as well as specific treatments [1]. Parents of children with health concerns are high users of online health information [6]. For example, 89% of parents of infants testing positive on a newborn screen accessed online information about the health concern, the majority of whom did so prior to meeting with their medical provider [7].

Disorders of Sex Development (DSD) are “congenital conditions in which the sex chromosomes, gonads, or external genitalia are considered atypical” [8]. DSD are subcategorized as Sex Chromosome DSD, 46,XY DSD and 46,XX DSD; each encompass multiple specific DSD conditions. In the aggregate, the incidence of DSD is estimated to range from 1:100 to approximately 1:4500–5000 live births (more precise incidence determination complicated by issues such as varying disorders being included in DSD incidence studies and variability in DSD nomenclature) [9–11], with some individual conditions quite rare. DSD are most frequently identified at birth because of atypical genital appearance or discordance between results of prenatal diagnostic testing and genital appearance at the time of delivery. In some cases, urgent medical care is needed at time of birth (e.g., classic congenital adrenal hyperplasia).

Parents of children with DSD often work with health care providers on difficult shared decision-making tasks such as choosing an infant’s initial gender assignment, discussing potential gonadectomy due to heightened cancer risk, and considering genitoplasty of atypical genitalia (historically, the standard of care). Infant genitoplasty is controversial; some DSD advocates strenuously argue against elective infant surgery while the vast majority of parents choose this option [12]. Parents of children with DSD describe significant distress during the diagnostic period, voice concerns about DSD-related stigma, report frustration related to perceived conflicting DSD information, and note stress over treatment decisions [13].

Given that DSD are relatively uncommon yet complex, parents of children with DSD conditions can benefit from high quality health information. Comprehensive education and full understanding of health conditions and treatment options are required for successful patient- and family-centered care and shared decision-making [14, 15]. Multiple aspects of web-based content determine its value (e.g. readability, understandability, actionability, accuracy of information, and reading level), and validated assessment tools have been created to gauge the quality of online health resources [16, 17]. There is a dearth of research specifically examining the quality of DSD online health information. One published article from nearly 20 years ago reported that much of the online information related to surgery and “intersex anomalies” failed to conform to pediatric surgical standards recommended at that time [18]. Another article included DSD in the category of “controversial” urologic conditions and reported that online information related to the “controversial” conditions was less accurate and less complete relative to the non-controversial conditions (e.g., renal cancer) [19]. A recent article focusing on just one DSD—hypospadias—and only evaluating treatment-related information determined that online material was of adequate quality but the reading level was high [20].

The DSD-Translational Research Network (DSD-TRN), funded by the National Institute of Health, was established in 2011 to standardize care based on current best practice recommendations, facilitate discovery of genetic causes of DSD, and create a clinical registry and biobank to promote research directly intended to improve clinical outcomes [21]. As members of the DSD-TRN Psychosocial Workgroup, we are invested in ensuring that DSD-TRN member sites are providing high quality DSD educational resources to a variety of stakeholders, including patients and families, advocates and care providers. In the present study, we systematically evaluated the quality of DSD-related online health care information presented by the 12 pediatric institutions comprising the DSD-TRN. We primarily focused on webpages identified by DSD-TRN sites as their “DSD Team Pages” (and links stemming from those webpages) to evaluate DSD-related information which consumers would presume had been vetted and approved by DSD specialists. We also conducted an independent search of DSD-TRN institution websites using key DSD terminology to review other institutional webpages that patients and families may locate that are not specifically affiliated with the DSD team. Based on previous studies in different populations [17, 22], we predicted that we would identify areas for improvement in the quality of information provided by our DSD-TRN sites.

## Methods

### Webpage identification and classification

**Fig. 1:**
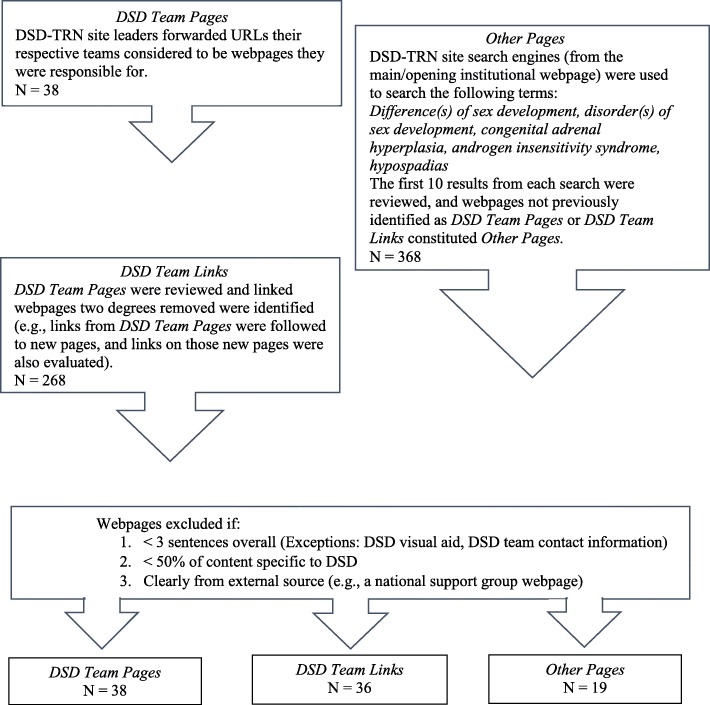
DSD webpage categories and identification process

We reviewed the websites of the 12 DSD-TRN member sites in June and July 2017 (Fig. 1). First, we contacted each site principal investigator with a request for the URLs their DSD team were responsible for (i.e., “DSD Team Pages”). Individual webpages were then examined for links to other institutional webpages. Webpages containing DSD-related content that were identified within two clicks from each team-identified URL were also evaluated (i.e., “DSD Team Links”). In addition, we searched common DSD terms using the search engine on each institution’s home page. The resulting webpages that had not been previously categorized as a “DSD Team Page” or “DSD Team Link” were categorized as “Other Pages.”

### Measures and coding process

We used three validated health information evaluation tools with standardized administration instructions to assess multiple dimensions of information quality, as well as one measure developed for this study (Table 1 here). The Simple Measure of Gobbledygook (SMOG) estimates the years of education a person needs to understand a piece of writing [23]. The Patient Education Materials Assessment Tool (PEMAT) [24], designed by the Agency for Healthcare Research and Quality, evaluates whether 1) healthcare information is understandable, termed “understandability” and 2) actions consumers can take related to the information are clearly outlined, termed “actionability”. A webpage is determined to be “understandable” if at least 70% of the 17 understandability items meet criteria. Similarly, a webpage is deemed to be “actionable” if at least 70% of the 7 actionability items meet criteria. The DISCERN is used to assess the quality of information related specifically to treatment decisions [25–27]. It is used to evaluate 1) the reliability and trustworthiness of material, and 2) whether specific details related to treatment options are presented in a balanced and complete manner. In addition, an overall quality rating is determined. We specifically examined information about two treatment options relevant to the DSD population: hormone replacement and surgery. Any webpage (DSD Team Page, DSD Team Link or Other Page) that had material related to either of these treatment options was identified and coded. A distinct DISCERN score was calculated for each treatment option.

**Table 1:** Measures used in study and scoring process

Tool	Validated	A measure of	Coding plan	Scoring
Simple Measure of Gobbledygook (SMOG)	Yes	Reading level (grade) of written material	All DSD Team Pages reviewed as one document, resulting in a single score DSD Team Links and Other Webpages scored individually	Count multisyllabic words across 30 sentences; sentences taken from beginning, middle and end of document Total sum converted into grade level
Patient Education Materials Assessment Tool (PEMAT)	Yes	Understandibility and actionability of information	All webpages (DSD Team Pages, DSD Team Links and Other Pages) scored individually Each webpage received both understandability and actionability score	2 scales: “understandability” (17 items), “actionability” (7 items) Rating: 0 (Disagree) or 1 (Agree) that information presented on a page met item criteria 70% items meeting criteria considered to be acceptable for each scale
DISCERN	Yes	Quality of online health information regarding specific treatment choices	Any webpages with information related to hormone replacement or surgery scored individually Each treatment received own DISCERN score	15 specific items + 1 “global” item Rating: 1 (criterion not met at all) to 5 (criterion completely met) Score (sum of 15 items)^a^ • < 38 = Poor • 39–50 = Fair • 51–62 = Good • 63–75 = Excellent
Completeness Rating	No	Overall completeness of information provided across a range of DSD topics	All DSD Team Pages and DSD Team Links reviewed individually Overall completeness score for site generated across webpages	25 items Rating: 0 (information not provided about item) or 1 (information provided about item) “Percentage complete” score for each site

^a^Based on Rao et al., 2012

Note: Prior to coding, coders reviewed each measure’s administration manual and/or scoring criteria. Coders practiced coding on DSD Team Pages of institutions that were not part of the DSD-TRN (found through a Google search), with discrepancies in coding resolved through review of manual and discussion under the supervision of the first two authors

Finally, we developed a 25-item “Completeness” measure to assess overall thoroughness of information based on domains we believed to be important for comprehensive understanding of DSD (e.g., terminology, etiology, condition management, psychosocial support). We solicited informal feedback on our items by contacting a convenience sample of leadership from DSD advocacy and support groups and asking them to score each item on a 5-point scale: “not important/don’t include” (scored “0”) to “very important” (scored “5”). Of the seven organizations contacted, six responses were received by the established deadline from leadership of four unique organizations. We ordered items by the frequency they were rated a 4 or 5.

Two coders with college-level education, but no advanced training specific to DSD, evaluated webpages using these tools. A primary coder (T.J.) evaluated 100% of the webpages, and this coder’s data were used in analyses. A second coder (A.S.) coded 30% of the webpages (randomly selected from within each of the DSD Team Pages, DSD Team Links and Other Pages categories) to determine interrater reliability and ensure standardized use of tools. Interrater reliability was determined by calculating percent agreement [28] and was excellent for the SMOG (90%), PEMAT (92%) and the Completeness rating (95%), with lower agreement between raters for the DISCERN (74%).

Descriptive statistics summarize findings of the health information quality assessment measures. This study was exempt from IRB review because human subjects data were not included.

## Results

### Webpage identification

A total of 674 unique webpages were reviewed; 93 webpages met inclusion criteria and were evaluated using the aforementioned health quality assessment tools. Ten of 12 sites identified at least one DSD Team Webpage. Of those 10 sites, the total number of webpages categorized as DSD Team Pages or DSD Team Links ranged from 1 to 11, with a mean (SD) of 7.4 (3.5) webpages (Table 2 here). Six of 12 sites had institutional webpages with DSD content that were not linked to DSD Team Pages (i.e., “Other Pages”). Of the 93 pages meeting inclusion criteria, 37 pages referenced either surgery or hormone replacement options (7 DSD Team Pages, 24 DSD Team Links, 6 Other Pages). Surgery-related content was found on 33 webpages and hormone replacement information on 20 pages, with some webpages containing information related to both treatments.

**Table 2:** SMOG, PEMAT, DISCERN & Completeness ratings for Team Pages (TP) and Team Link (TL) webpages

Site	Webpage	Webpage Description	SMOG	PEMAT		DISCERN		COMPLETE-NESS
				Understandability	Actionability	Hormone	Surgery	
			Grade Level^a^	%	%	max = 75	max = 75	%
A	TP 1	Introduction: DSD clinic	16	62	20			48
	TP 2	DSD Treatments		75	20			
	TP 3	DSD Types		69	20			
	TP 4	What to expect at first DSD Clinic appointment		62	20			
	TL1	Health topic: CAH	18	62	20			
B	TP 1	Introduction: DSD team	14	69	40			52
	TP 2	DSD detection and diagnosis		67	0			
	TP 3	DSD treatments		69	40	39	43	
	TL 1	DSD research study recruitment	14	69	40			
	TL 2	Health topic: CAH	13	69	20	31	19	
	TL 3	Health topic: Hypospadias	14	69	40	32	31	
	TL 4	Introduction: DSD program	15	69	20			
	TL 5	Overview: DSD and Gender program	17	81	33			
C	TP 1	Overview: DSD Clinic	16	67	0			40
	TP 2	DSD Types		69	0			
	TP 3	DSD Treatments		67	0	23	28	
	TP 4	What to expect at first DSD Clinic appointment		67	0			
	TP 5	DSD clinic contact information		62	40			
	TL 1	Overview: DSD	13	69	0	19	18	
	TL 2	Health topic: AIS	13	69	0	32	25	
	TL 3	Health topic: CAH	12	69	40	34	31	
	TL 4	Health topic: Hypospadias	12	75	40		35	
	TL 5	Health topic: MGD	11	69	20	26	26	
D	TP 1	Overview: DSD program	17	69	40		18	Not coded^b^
	TP 2	DSD types and treatment		70	0			
	TP 3	DSD Clinic Contact Information		62	20			
	TP 4	Health topic: DSD and Ambiguous genitalia		69	20	22	26	
	TP 5	Health topic: Cloacal malformations		62	20			
	TP 6	Health topic: CAH		62	40			
	TP 7	Health topic: Gonadal dysgenesis		62	20			
	TP 8	Health topic: Vaginal agenesis		69	20		38	
	TL 1	Overview: DSD program	18	67	0			
	TL 2	Overview: CAH care center	17	69	40			
	TL 3	FAQ: CAH	14	69	0	42	32	
E	TP 1	DSD Resources	16	56	0			40
	TP 2	Overview: DSD clinic		69	20			
	TP 3	Health topic: Ambiguous genitalia and Gender determination		69	0			
	TP 4	Overview: DSD and Gender program		69	20			
	TL 1	Health topic: Micropenis	14	69	0	29		
	TL 2	Health topic: Hypospadias	14	69	20		23	
	TL 3	Health topic: Ambiguous genitalia and DSD	14	69	0	19	23	
F	TP 1	Overview: DSD and DSD Clinic	15	69	0			56
	TP 2	Overview: DSD and DSD Clinic, DSD Resources		69	40			
	TP 3	DSD Types		67	0			
	TL 1	Health topic: Turner Syndrome	10	62	0			
	TL 2	DSD decision-making checklists	17	44	0			
	TL 3	CAH decision making checklist	9	69	60			
	TL 4	Hypospadias decision-making checklist	9	81	50		33	
	TL 5	MRKH decision-making checklist	9	69	60		39	
	TL 6	Overview: DSD	11	69	0	17	19	
	TL 7	Health topic: CAH (newborn)	12	75	33	40	34	
	TL 8	Health topic: CAH	11	75	33	36	36	
G	TP 1	Overview: DSD program	13	85	40			40
	TP 2	DSD Clinic Contact Information		50	40			
	TP 3	What to expect at first DSD Clinic appointment		60	20			
	TP 4	DSD Resources		62	20			
	TL 1	DSD detection and diagnosis	13	69	40		26	
	TL 2	Health topic: Hypospadias	10	81	33		40	
	TL 3	Health topic: MRKH	11	69	40		38	
	TL 4	Health topic: Turner Syndrome	14	69	60	33	27	
	TL 5	Health topic: Hypospadias repair	9	69	60			
	TL 6	Reproductive organs images		69	20			
	TL 7	Decision-making checklist	8	77	60			
H	TP 1	Overview: DSD clinic	16	69	80			20
	TL 1	Health topic: DSD	13	69	0	18	31	
	TL 2	Health topic: Hypospadias	14	69	0		25	
I	TP 1	Introduction: DSD and DSD team	15	67	20			36
	TP 2	What to expect at first DSD Clinic appointment		69	40			
	TP 3	DSD Types		70	40			
	TP 4	DSD Resources		69	0			
	TP 5	DSD Research Studies		69	20			
	TL 1	Glossary: DSD terms	13	69	20		27	
	TL 2	Glossary: DSD tests	15	54	20	33	28	
J	TP 1	Overview: DSD and DSD Program	17	69	40			12
		Mean	13.5	67.9	23.5	29.2	29.3	38.2
		SD	2.6	6.2	19.7	8.0	7.0	14.3
		Minimum	8	44	0	17	18	12
		Maximum	18	85	80	42	43	56

^a^Grades 13–16 indicate college level education needed to ensure complete comprehension and 17–18 reflect graduate training education necessary

^b^Site not coded because webpages changed prior to completeness ratings

### Quality results for DSD team pages and DSD team links

SMOG readability scores of DSD Team Pages ranged from 13 to 17 with a mean (SD) of 15.5 (0.7), representing college reading level (Table 2). Similarly, readability of content on DSD Team Link Webpages were also written at a high grade level, with a mean (SD) of 12.9 (2.6) and range of 8–18.

**Fig. 2:**
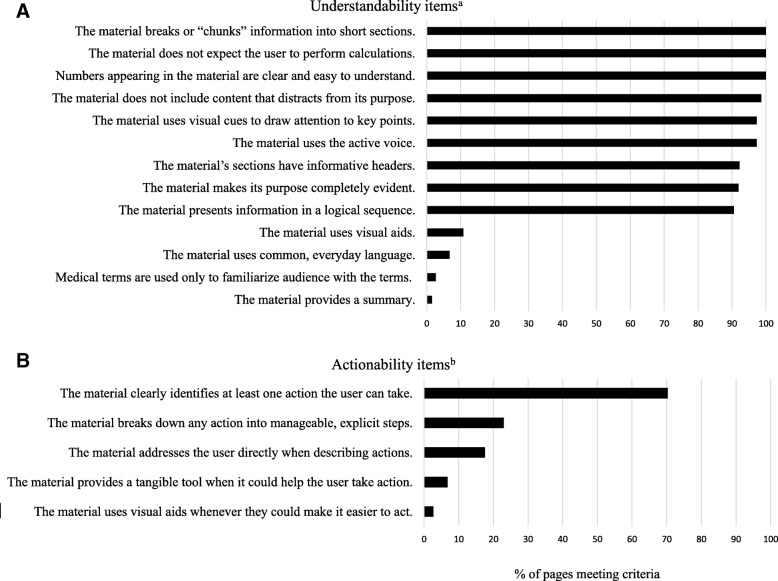
The percentage of DSD Team Pages and Team Links that satisfied criteria on PEMAT items

The mean (SD) PEMAT understandability score for individual DSD Team Pages and Team Links was 68% (6%) of criteria met; scores ranged from 44 to 85% criteria met, and only 12 of the 74 webpages met or exceeded the understandability cut-off (i.e., ≥ 70% of the PEMAT criteria). There was little variability between sites in PEMAT understandability ratings. Averaging the understandability scores for each site’s DSD Team Pages and Team Links resulted in a range of mean understandability scores of 66–70% for the 10 sites. As illustrated in Fig. 2a, the majority of webpages (> 90%) met criteria for the top nine understandability items (e.g., chunking information) whereas relatively few webpages (< 15%) met criteria for the final four applicable criteria.

PEMAT actionability scores were lower compared to understandability. Twenty-two of the 74 DSD Team Pages and Team Links had a PEMAT actionability score of 0% (i.e., no actionability), one webpage had a score of 80%, and the remaining webpages ranged from 20 to 60%. Overall mean actionability was 23% (20%), with a median of 20% actionability criteria met. Averaging each sites’ actionability percentages for the DSD Team Pages and Team Links resulted in site mean scores ranging from 9 to 40%. The item most likely to meet PEMAT actionability criteria was “having at least one action that could be taken” (Fig. 2b). The most frequent action identified was related to contacting the team for information or for scheduling an appointment, and the next most frequent action specified how to find more information related to DSD conditions. Examples of other actions included how to share information with children, how to use a checklist for decision-making, when to call the doctor in case of emergency, and how to participate in research.

**Fig. 3:**
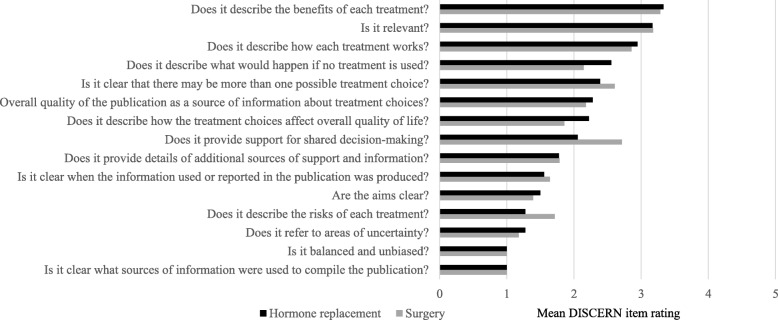
Mean DISCERN item ratings for Hormone treatment and Surgery for DSD Team Pages and Team Links that had treatment information (0–5 maximum)

DISCERN scores demonstrated inadequate quality of treatment-related information. Hormone replacement information was provided by eight different DSD-TRN sites across 18 DSD Team Pages and Team Links, with a mean (SD) DISCERN score of 29.2 (8.0), representing “poor” quality. Surgery-related information was found on 28 DSD Team Pages and Team Links, with a mean (SD) DISCERN score of 29.3 (7.0), also representing “poor” quality. Mean DISCERN scores for hormone replacement and surgery-related information generally were similar for a given site (Table 2); for seven of the eight sites, the difference between the mean hormone replacement and mean surgery DISCERN score was less than 5.5 points. For both hormone replacement and surgical information, webpages performed best in describing benefits of treatment, highlighting treatment relevance, and describing how treatments work, but performed poorly in referencing sources of information, providing balanced and unbiased information, and referring to areas of uncertainty (Fig. 3).

**Fig. 4:**
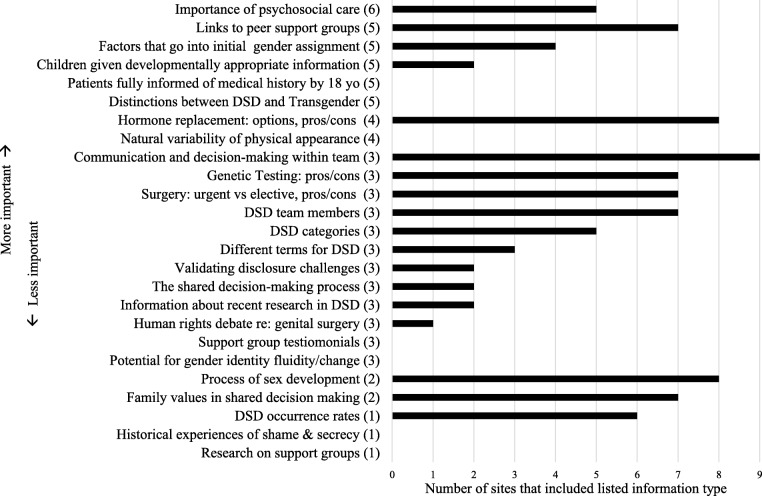
Number of sites (out of 9) that included listed DSD information on DSD Team Pages or Team Links, rank ordered according to number of stakeholders who assigned highest rating of importance

In terms of Completeness ratings, sites ranged from including 12–56% of the 25 items, with a mean (SD) completeness score of 38% (14%). As shown in Fig. 4, sites most consistently provided information related to DSD team functioning, causes, prevalence and treatments for DSD, and psychosocial care (including links to support groups). Several items frequently rated as “important” by patient advocates were rarely presented (e.g., how gender assignment occurs) or not at all (e.g, importance of patients knowing complete medical history by age 18 years; distinctions between DSD and transgender; natural variability in physical appearance).

### Quality results for other pages

The quality evaluation results for the Other Pages were comparable to the DSD Team Pages and Team Links. The mean SMOG score for these pages was 14.3 (2.7). PEMAT understandability and actionability scores of Other Pages were both below recommended cut-offs; mean (SD) scores were 62.9 (9.3) and 18.8 (23.5), respectively. Two Other Pages had information related to hormone replacement in DSD; DISCERN scores for these webpages were 17 and 29, both in the “poor” quality range. Five Other Pages had information related to surgery and DSD; mean DISCERN score was 28.4 (5.8), also falling in the “poor” quality range.

## Discussion

Our audit of webpages associated with DSD-TRN member sites demonstrated unsatisfactory quality of the health education material. Across three standardized measures, webpages consistently failed to meet established criteria. Reading level across all webpages was higher than the 4th–6th grade level recommended for health education material [29, 30]. The majority of the webpages also did not meet either PEMAT understandability or actionability criteria, and treatment-related information (for both hormone replacement and surgery) were rated poor quality applying DISCERN criteria. The majority of sites included fewer than 50% of items we had determined to be important to ensure that “complete” information is provided and often did not include information that patient advocates identified as “important.” The suboptimal provision of health information related to DSD has significant ramifications for all stakeholders. When diagnosis occurs in infancy, parents are expected to participate in decision-making that can have irrevocable implications for their children’s medical and psychosexual futures. When diagnosis occurs in later childhood, or even adulthood, patients must make sense of complex information that can initially cause significant distress and impede careful consideration of treatment options. Indeed, many adult patients with DSD report inadequate understanding of their own medical history and poor satisfaction with their level of knowledge [31].

Some strengths were noted in the evaluated online information. For example, webpages tended to present focused information in chunks, displayed in a logical sequence (using headers) and required minimal calculations. In addition, information related to both hormone replacement and surgical options was provided by all sites, with an emphasis on the mechanisms and benefits of treatment. However, several areas of improvement were identified. As a first step, reducing reading level by simplifying word choice and eliminating medical jargon when possible should aid in understandability. Use of visual aids could enable readers to better understand concepts related to DSD, such as internal anatomy and typical sexual development. In addition, detailing specific actions that patients or caregivers can take may assist families in coping and expanding their knowledge base. For examples, families can be encouraged to have conversations within their social networks to educate those caring for their child and elicit social support, and be encouraged to contact other patients with a DSD or their caregivers for peer support [8]. Patient (or proxy) decision-making may be enhanced by greater acknowledgement of the risks of different treatment options and acknowledging areas of uncertainty [32]. It is critical that health care teams be aware of the possibility that families may access DSD information on institution webpages that is not connected directly to the multidisciplinary team. Although these sites may not be directly vetted by DSD health care providers, patients/families may assume that they are. Finally, we reviewed websites from the perspective of information provided to adult patients or parents of children with DSD, and in fact none of the websites provided information directly targeting children or adolescents. Content that targets patients with DSD across the developmental span is needed.

Our findings must be interpreted within the limitations of our approach. First, website content is fluid– it is possible that since our analysis, institutional websites have been updated, rendering some of our findings out-of-date. It is also possible that had we used different search words, we may have identified additional Other Pages. While we did involve patient advocates in identifying high-priority items for our completeness evaluation, a more systematic evaluation of patient stakeholders perspectives from a variety of DSD conditions may identify different priorities for online content, as has been found in other populations [33]. Additionally, our interrater reliability for the DISCERN was lower than for the other measures; findings related to this tool should be interpreted with greater caution. Finally, there are a number of features of health information quality that we did not evaluate, including cultural sensitivity and privacy of data collection [22] – these aspects could be explored in future studies and should be considered in online content creation or revision.

## Conclusions

The poor quality of DSD content on these webpages are in line with findings from other analyses of the quality of online health information [16, 17, 22], including studies that focus primarily on academic medical centers [34]. The imperative for complete and understandable information is heightened for conditions in which aspects of diagnosis and/or care are controversial. Consumers seek online health information even if instructed to not do so by their providers [7]; to assume otherwise is to be naïve and fail to provide expected standards of patient/family-centered care. Recently, the online content on one of the DSD-TRN member websites was referenced negatively during proceedings for the California Senate Concurrent Resolution 110 which proposes to prohibit genital surgery in DSD conditions “until a time at which an individual can participate in the decision” [35]. Providing carefully vetted and comprehensive health information which follows recommended standards for health information quality is essential, and vigilance over the content and quality of online information is warranted.

The initial information that patients and families receive about DSD can set the course for coping and adjustment during the diagnostic process [13, 36]. Given that families may be informed of a possible DSD diagnosis by medical providers who are not specialists in the care of these conditions, it is imperative that online information serves to demystify and destigmatize these conditions so that families’ early distress and confusion do not challenge longer-term values during decision-making. To that end, we have provided site-specific feedback to member institutions of the DSD-TRN to highlight the need for website revisions and identify strengths and weaknesses of individual site health material. In addition, the DSD-TRN can use these health literacy measures and the data presented herein to guide development of “best practice” DSD health content that can be standardized to be used across DSD-TRN member sites. Tools such as those utilized in the current study can strengthen such health literacy quality improvement initiatives.

## Abbreviations

DSD: Disorder(s) of sex development
DSD-TRN: DSD-Translational Research Network
PEMAT: Patient Education Materials Assessment Tool
SMOG: Simple Measure of Gobbledygook
